# Affective States During the First Wave of the COVID-19 Pandemic: Progression of Intensity and Relation With Public Health Compliance Behavior

**DOI:** 10.3389/fpsyg.2022.883995

**Published:** 2022-07-07

**Authors:** Yanick Leblanc-Sirois, Marie-Ève Gagnon, Isabelle Blanchette

**Affiliations:** ^1^Départment of Psychology, Université du Québec à Trois-Rivières, Trois-Rivières, QC, Canada; ^2^School of Psychology, Université Laval, Québec, QC, Canada

**Keywords:** affective state, COVID, fear, public health, pandemic

## Abstract

The COVID-19 pandemic was expected to cause intense affective reactions. This situation provided a unique opportunity to examine the characteristics and correlates of emotions in a real-world context with great significance. Our study aimed to describe the progression of positive and negative affective states during the pandemic, and to investigate which affective states predicted compliance with public health measures. We undertook a survey of affective states in the province of Quebec at the beginning, the peak, and the aftermath of the first wave of the COVID-19 pandemic. We recruited 530 responders; 154 responded to all three surveys. We used self-report scales to measure affective states and compliance with public health measures. We then computed separate linear regressions for the three phases of our study, with compliance with health measures as the dependent variable. Affective states were generally most intense at the beginning of the pandemic. Fear-related pandemic-related affective states reliably predicted compliance with public health measures in the three phases of our study. Positively valenced affective states related to the societal response also contributed predictive value, but only at the peak of the first wave.

## Introduction

On 11 March, 2020, the World Health Organisation announced that the spread of the novel coronavirus SARS-CoV-2 had given rise to a global pandemic. Governments across the globe implemented emergency public health measures to slow down the spread of COVID-19. In the province of Quebec, Canada, schools and universities were shut down on 13th March. Additional emergency measures were deployed in the following days and weeks as the number of new daily cases rose to a peak in the middle of April, followed by a peak of daily coronavirus-related deaths at the beginning of May (Institut national de la santé publique du Québec, 2021a). Lockdown measures were then gradually eased as the first wave of the pandemic waned. For instance, outdoor meetings with people from multiple households were allowed again on 23rd May and indoor meetings were authorized on 15th June, under certain conditions (Institut national de la santé publique du Québec, 2021b).

Events which negatively affect a large part of a local population, such as natural disasters (see Beaglehole et al., 2018, for a meta-analysis), tend to cause negative affective states in those who are affected by the event. Such a surge in the intensity of negative emotions could also be expected in the context of the COVID-19 pandemic. However, attempts to promote positive feelings of social solidarity and hope were also common at the beginning of the pandemic (Cheng et al., 2020), with the expectation that these affective states could help improve compliance with public health measures (Heffner et al., 2021). For this reason, we sought to further understand the public’s positive and negative affective reactions toward the pandemic during different phases of its first wave in the province of Quebec. We concentrated on two aspects of the affective response to the COVID-19 pandemic: the progression of the intensity of affective states during the first wave of the pandemic and the relation between affective states and compliance with public health measures.

### Progression of the Intensity of Affective States and the COVID-19 Pandemic

There is considerable uncertainty about whether affective states become less intense or more intense over time as people experience a sustained highly emotional event, such as the COVID-19 pandemic. Studies on people who have experienced large-scale disasters are not highly informative in the context of the COVID-19 pandemic as they focus on circumscribed events that occur over a relatively short period of time. Studies have mostly concentrated on psychological distress in the aftermath of a disaster (i.e., Beaglehole et al., 2018), not on the intensity of affective states during a disaster. Research published about the COVID-19 pandemic at the time of writing also documents the presence of positive (Bhat et al., 2020) and negative (Papkour and Griffiths, 2020; Schimmenti et al., 2020) pandemic-related emotions, but do not offer accounts of how their intensity or nature varied over time.

Previous empirical studies of the general progression of the intensity of affective states have shown that the duration of an emotion correlates positively with the initial intensity of the eliciting events (Verduyn et al., 2009). Moreover, longer-lasting emotions are elicited by a reappearance of the event or rumination about the event, longer-lasting events, and events of high importance (Verduyn et al., 2015; Verduyn and Lavrijsen, 2015). Together, these studies suggest that affective reactions to the COVID-19 pandemic can be expected to be long-lasting. However, these studies do not provide insight about the moment when the intensity of emotions can be expected to peak. Moreover, these studies relied on retrospective reports of remembered emotions that may not accurately reflect how intensely emotions were experienced at the time of the event (Levine and Bluck, 1997).

It is possible that the intensity of affective states evoked by the pandemic peaks close to the pandemic’s onset and decreases steadily afterward. Supporting this point of view, Frijda (1988) proposed laws of emotion include a rule of habituation, according to which emotions tend to become less intense with repeated exposure to the eliciting event. Following the rule, some studies obtained evidence of an early peak for the intensity of negative emotions. Betini et al. (2021) found a decline in depressed moods using questionnaires across four time points during the first wave of the pandemic in a Canadian sample, from mid-April to the end of July. Fancourt et al. (2021) also investigated self-reported anxiety and depression symptoms in the first 21 weeks after the onset of lockdown and found a decline in these symptoms during the first few weeks, suggesting that the intensity of negative affective states may also have declined.

However, it is also possible that the intensity of affective states related to the pandemic could remain stable as long as the pandemic was ongoing and lockdown measures were in place. Supporting this point of view, Frijda (1988) proposed laws of emotions also includes a rule of apparent reality, which states that the intensity of emotions should be proportional to the degree to which the events elicited by the emotions appear or are appraised as real. In the context of a pandemic, the apparent reality of a situation may, for instance, be related to the number of active cases and deaths, or it may be related more closely to the intensity of ongoing lockdown measures at a given time. Both of these interpretations suggest that the intensity of affective states should be high at the peak of the pandemic, when the number of cases was high and lockdown measures were still in place. There is also support in the literature for stability across time in the intensity of the affective states evoked by the pandemic. Martín-Brufau et al. (2020) measured mood during the first 2 weeks of the pandemic in a Spanish sample and found no trend for a decrease in time. However, this is a relatively short time frame. Contrary to the studies cited previously, several studies conducted during the early months of the COVID-19 pandemic found relatively stable levels of anxiety and depression across time (Hyland et al., 2020; McPherson et al., 2021). However, mental health symptoms, not affective states, were the object of these studies.

The progression of affective states may also depend on their valence. Supporting, this third point of view, a study using automatic sentiment analysis of social media posts showed a decrease in negatively valenced pandemic-related expression and an increase in positively valenced expression as the pandemic progressed (Zhao et al., 2020), consistent with an early peak for the intensity of negatively valenced affective states. Another study using automatic sentiment analysis suggested that mean indicators of mood worsened immediately after lockdown announcements, but recovered relatively quickly (Kruspe et al., 2020). One limit of these studies is that they measure the valence of messages posted on social media, not experienced affective states as reported by participants (Mohammad, 2017). Notably, a positivity bias has previously documented in the expression of emotions on these platforms (Reinecke and Trepte, 2014).

Thus, evidence on the progression of the intensity of the affective states during the early phases of the COVID-19 pandemic are mixed. Moreover, some of this evidence relies on automatic sentiment analysis of social media posts which may not reflect experienced affective states (Mohammad, 2017), notably because a positivity bias has previously documented in the expression of emotions on these platforms (Reinecke and Trepte, 2014). Several studies also offered a somewhat incomplete portrayal of the progression of the intensity of affective states by not including measures collected at the beginning, peak and end of the first wave of the COVID-19 pandemic. Finally, most researchers have investigated mental health symptomatology, rather than affective states themselves.

### Do Affective States Predict Compliance With Public Health Measures?

The societal response to the COVID-19 pandemic has relied on the public’s compliance with public health measures to slow down the spread of the virus. Yet, at the beginning of the pandemic, little was known about the individual factors that predict compliance with public health measures. Research has since revealed some predictors of higher compliance with public health measures during the pandemic, notably older age (Brouard et al., 2020; Haischer et al., 2020; Solomou and Constantinidou, 2020) and female gender (Clark et al., 2020; Galasso et al., 2020; Haischer et al., 2020; Solomou and Constantinidou, 2020; Nivette et al., 2021). Higher self-efficacy (Clark et al., 2020; Hamerman et al., 2021; Jørgensen et al., 2021), higher perceived vulnerability to disease (De Coninck et al., 2020; Hromatko et al., 2021), higher trust in science (Hromatko et al., 2021; Plohl and Musil, 2021), prosocial personality traits (Dinić and Bodroža, 2021; Nivette et al., 2021; Ścigała et al., 2021), and lower exposure to misinformation (Roozenbeek et al., 2020; Simonov et al., 2020; Greene and Murphy, 2021; Lin, 2022) have also been linked to higher compliance with public measures. We aimed to investigate whether pandemic-related affective states also predicted compliance with public health measures. Moreover, we aimed to determine whether the same affective states would be predictive of compliance with public health measures during the different phases of the first wave of the COVID-19 pandemic.

The approach/avoidance motivation model of emotions provides a theoretical framework which proposes causal effects of emotions on behavior. According to this model, emotions can influence behavior by providing motivation toward approaching or avoiding real or anticipated stimuli (Elliot et al., 2013). Positive emotions are generally linked to an approach motivation, while most negative emotions, with the notable exception of anger, are generally linked to an avoidance motivation. This model suggests that negatively valenced affective states such as fear and anxiety could increase avoidance of COVID-19 and may thus lead to more compliance with public health measures. At the same time, positively valenced affective states evoked by the societal response (i.e., the way governments, public health organizations and individuals reacted toward the pandemic) could increase active participation in this response and specifically encourage compliance with public health measures.

Supporting these assumptions, higher levels of fear evoked by COVID-19 were a strong predictor of more behavior change during the COVID-19 pandemic (Harper et al., 2021). Moreover, a higher intensity of positive affective states evoked by the societal response at the beginning of the pandemic predicted higher levels of compliance with public health measures (Bogg and Milad, 2020). However, these previous studies only investigated the links between behavior and affective states during one phase of the pandemic. Therefore, the temporal characteristics of the link between affective states and behavior throughout the pandemic have yet to be studied.

### Objectives

The first objective of the study was to document the progression of affective states across different phases of the first wave of the COVID-19 pandemic. We aimed to determine whether the progression of the intensity of affective states would follow a downwards trend as the pandemic unfolded, or whether the intensity of affective states would remain relatively stable across different phases of the first wave of the pandemic.

The second objective of the study was to document the predictors of compliance with public health measures during the pandemic, and specifically to explore the predictive value of self-reported affective states. We hypothesized that two groups of affective states would be associated with higher compliance with public health measures: negative affective states evoked by the pandemic and positive affective states evoked by the societal response.

## Materials and Methods

### Participants

One week after emergency measures were declared in the province of Quebec in response to the COVID-19 pandemic (13th March), we recruited a convenience sample on social media platforms for an online survey about behavior, affective states, reasoning, and mental health in the context of the COVID-19 pandemic. Participants were over 18 years of age, were fluent in French, and lived within the borders of the province of Quebec. Between 20th March and 27th 2020, 530 responders (19.2% men, 79.2% women, 1.6% other/no response; M = 35.3, SD = 13.4) completed the first questionnaire.

**Figure 1:**
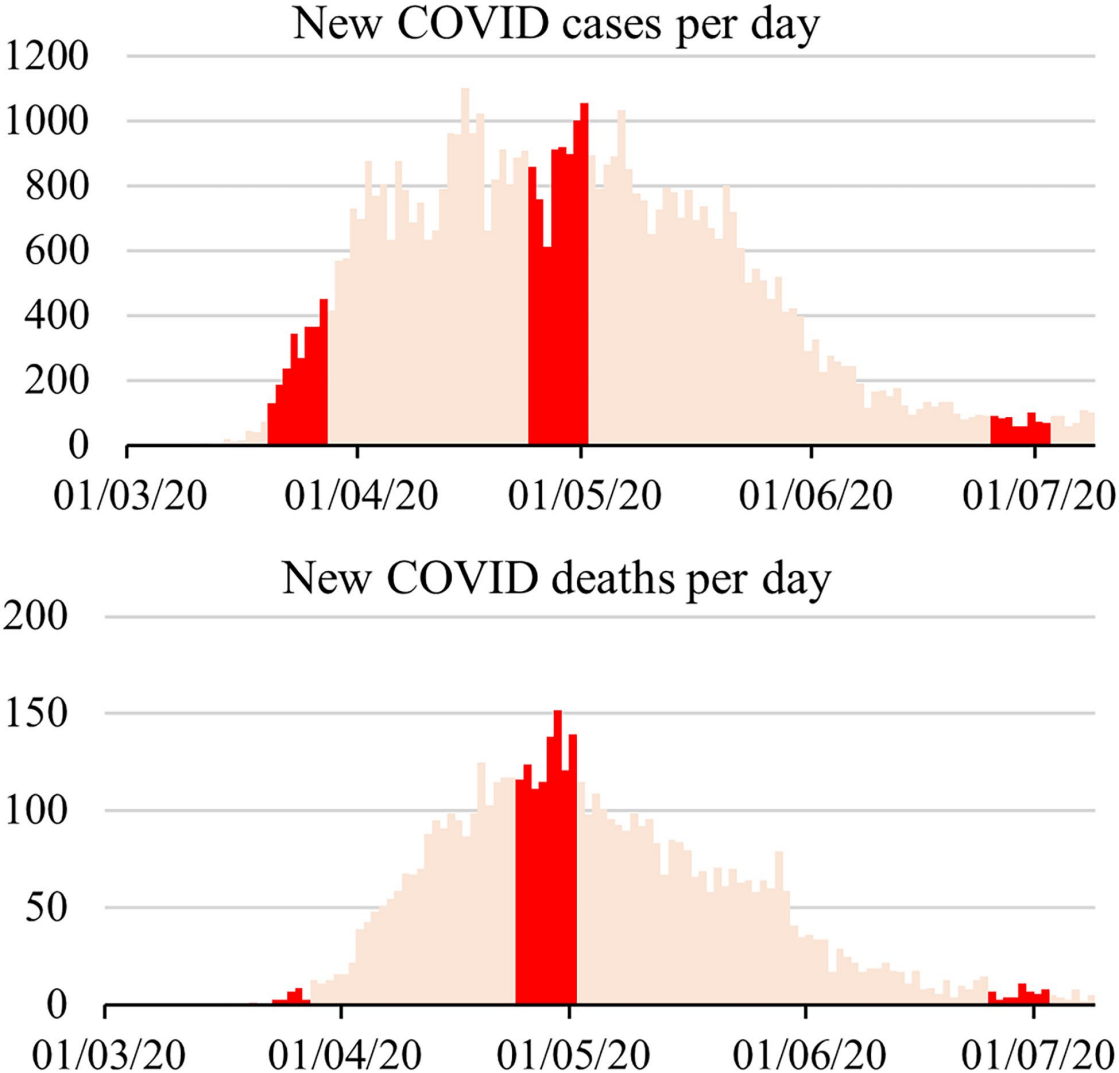
New COVID-19 cases and deaths per day in the province of Quebec, Canada. Timings for phases 1, 2, and 3 of the study are identified in darker color. Data obtained from Institut national de la santé publique du Québec (2021a). Dates are provided in day/month/year format.

Of these, 224 responders (28.7% men, 71.3% women; M = 37.8, SD = 13.9) filled the second questionnaire between 24th April and 1st May, when the number of new cases per day in Quebec was close to its maximum. A third questionnaire, accessible between 26th June and 3rd July, received 171 responses from the responders who had filled the first questionnaire (25.1% men, 74.9% women; M = 37.9, SD = 13.9). In total, 154 responders filled out all three questionnaires (29% of total). Participants did not receive financial compensation. See Figure 1 for information about the state of the pandemic in the province of Quebec during the three phases of the study.

Eighty-seven percent of the phase 1 sample reported that the pandemic had affected their work or studies, either by changing the conditions in which the work took place or by interrupting work. Seventy-two percent reported that they had put some leisure activities on hold due to the pandemic, 29% reported financial difficulties due to the pandemic, and 90% reported that their social life was constrained by the pandemic. In phase 2, 92% of participants reported seeking information about the pandemic at least three times per week. These data confirmed that the pandemic affected the life of most of our responders in an important way.

The research project was approved by the Human Subject Research Ethics Committee at Université du Québec à Trois-Rivières under certificate CER-20-266-10.03. Informed consent was obtained at the beginning of the online questionnaire with a letter of information stating the objectives, duration, and inclusion criteria for the study. Contact information for the research team was available on this letter in case potential participants had questions about the study. Participants provided consent after reading the letter of information.

### Measures

All following measures were presented in an online experiment programmed with Qualtrics (Qualtrics, Provo, UT). An English translation of the full questionnaire can be found on the project site at https://osf.io/gb2mq/?view_only=63f5e415f08c41d6b16d195f73b3db96. For purposes of brevity and clarity, we limit our detailed description of measures to those associated with the research question introduced above.

#### Sociodemographic Information

We asked participants to provide their age, gender, and level of education during phase 1 of the study.

#### Affect Questionnaire

During phases 1, 2, and 3 of the study, we collected information about participants’ affective states. We asked participants to indicate, on a seven-point Likert scale (1 = very weak, 7 = very strong), the level of intensity of seven affective states attributed to COVID-19: fear, anger, disgust, hope, anxiety and shock, as well as their feeling of security. We also asked participants to indicate the level of intensity of these seven affective states evoked by the societal response to COVID-19. Specifically, we asked the following question: “Please indicate at which level of intensity you currently feel the following emotions, in relation to the response of the Quebecois, of public health organizations and of the government toward COVID-19.” Additionally, we asked participants about three more affective states related to the societal response: solidarity, pride, and satisfaction. Additionally, we obtained scores for frustration and solitude in phases 2 and 3 of the study. Self-reported affective states were obtained during phases 1, 2, and 3 of the study.

#### Behavior Questionnaire: Compliance With Public Health Measures

We provided participants with a list of 12 behaviors that could be adopted in response to the COVID-19 pandemic and asked them to indicate which behaviors they adopted. In the first questionnaire, we asked about the presence or absence of behaviors. In phases 2 and 3, the questionnaire was adjusted to measure the frequency of the same behaviors during the previous 2 weeks (phases 2 and 3) on a scale of 1—Never to 4—Often.

Three of the listed behaviors corresponded specifically to public health measures that were strongly encouraged by local authorities from the first week of the pandemic to the end of its first wave: washing hands often, avoiding touching one’s face, and social distancing. We created a score of compliance with public health measures by adding presence/absence scores (phase 1) or frequency scores (phases 2 and 3) for these three behaviors. A higher score represented more compliance with public health measures.

### Statistical Methods

#### Dimension Reduction

To limit the number of statistical analyses, we first ran a principal components analysis on affective states associated with the pandemic on data from all three phases together (*N* = 925) in order to identify clusters of related affective states. The analysis was run in SPSS 24. Prior to running this data reduction procedure, we confirmed that the Kaiser–Meyer–Olkin test yielded a value of 0.74 and that Bartlett’s sphericity test was significant (*p* < 0.001), indicating that the data were suitable for a principal components analysis. For each set of responses to the affective state questionnaire collected during phases 1, 2, and 3 of the study, we derived a component score for each conserved component with the regression method.

Following the separation into principal components, a varimax rotation was used and all components with eigenvalues above 1 were conserved. Scores for each component that had an initial eigenvalue superior to 1 were then computed for each data point with the regression method, for the purposes of investigating the progression of these components across time and statistical links between these components and compliance with public health measures.

We also ran a principal components analysis with identical parameters on affective states associated with the societal response. Prior to running this analysis, we confirmed that the Kaiser–Meyer–Olkin test yielded a value of 0.83 and that Bartlett’s sphericity test was significant (*p* < 0.001), indicating that the data were suitable for a principal components analysis. For each set of responses to the affective state questionnaire collected during phases 1, 2, and 3 of the study.

#### Selective Attrition

Due to high attrition, we investigated differences between participants who completed all three phases of the study (*n* = 154) and those who did not (*n* = 376) in order to identify factors which might explain the decision to continue participating in the current study. More specifically, independent samples *t*-tests were conducted with length of participation as the independent variable. Dependent variables were age, gender, phase 1 score of compliance with public health measures, and phase 1 scores for all affect components extracted in the dimension reduction procedure. We also used a Mann–Whitney *U* test to study differences between complete and partial responders in terms of level of education.

#### Progression of Affective States

To investigate the progression of the intensity of affective states, we computed repeated measures ANOVAs on all component scores derived from the principal component analyses, with phase (1, 2, 3) as the independent variable. This analysis was done only on data from participants who responded to our questionnaire in all three phases of the study (*n* = 154). A Greenhouse–Geisser correction was used when Mauchly’s sphericity test was significant. Uncorrected degrees of freedom are reported below. For analyses showing a significant effect of phase, we used paired samples *t*-tests to identify significant differences between the three phases. A Bonferroni correction was used during these *post-hoc* comparisons: we reported results with *p* < 0.017 exclusively. We also investigated linear contrasts.

#### Prediction of Compliance With Public Health Measures

To explore links between affective states and public health measures during each phase of the pandemic separately, we first ran three separate multiple linear regressions on data from phases 1, 2, and 3 of the study. For each phase, a first regression model was built with only age, gender, and level of education as predictors. In a second step, component scores derived from measures of affective states were entered into a second model. We repeated this comparison of two models for each of the three phases of our study. We used phase 1 affective states to predict compliance with public health measures in phase 1, phase 2 affective states to predict compliance with public health measures in phase 2, and phase 3 affective states to predict compliance with public health measures in phase 3. Prior to interpretation, we confirmed that multicollinearity was not high: for all models, the variance inflation factor was below 2 for all predictors in all three regressions. Listwise exclusion was used for missing data.

## Results

### Dimension Reduction: Affective States

The principal components analysis on affective states related to the pandemic yielded three components explaining 70% of the variance. Item weights on the three components can be found in Table 1. We labeled our three factors “fear-related,” “other negative,” and “positive.”

**Table 1:** Labels and item weights for components extracted in separate principal component analyses for affective states related to the pandemic and related to the societal response.

	Related to the pandemic			Related to the societal response	
	Fear-related	Other negative	Positive	Positive	Negative
Fear	**0.807**	0.187	−0.200	−0.092	**0.826**
Anxiety	**0.781**	0.103	0.033	−0.042	**0.825**
Hope	0.080	−0.107	**0.908**	**0.736**	−0.139
Feeling of security	−0.504	−0.023	**0.663**	**0.680**	−0.304
Anger	0.223	**0.790**	−0.138	−0.333	**0.697**
Disgust	0.127	**0.856**	−0.004	−0.309	**0.656**
Shock	**0.730**	0.201	−0.040	0.091	**0.721**
Solidarity	nm	nm	nm	**0.797**	0.090
Pride	nm	nm	nm	**0.873**	−0.017
Hope	nm	nm	nm	**0.792**	−0.237

For descriptive purposes, we highlighted components > |0.6| in bold. nm, not measured.

The principal components analysis on affective states related to the societal response yielded two components explaining 62% of the variance. Item weights on the two components can be found in Table 1. We labeled our two factors “positive” and “negative.”

### Evaluation of Selective Attrition

Participants who completed all three phases differed from others on a number of variables of interest. Those who participated in all three phases were generally older, M = 38.0, SD = 14.1, than those who did not, M = 34.2, SD = 13.0, *t*_(523)_ = 2.98, *p* = 0.003, *d* = 0.279. They also obtained higher component scores for positive affect related to the societal response, M = 0.404, SD = 0.798, than those who ended their participation early, M = 0.222, SD = 0.947, *t*_(516)_ = 2.07, *p* = 0.039, *d* = 0.21, and had a higher level of education, U(N_stay_ = 154, N_leave_ = 373) = 21,830, z = −4.52, *p* < 0.001. No other differences were observed.

### Progression of the Intensity of Affective States

Fear-related affective states related to the pandemic was affected by phase, *F*_(2,298)_ = 114.61, *p* < 0.001, $n_{p}^{2}$ = 0.435. *Post-hoc* comparisons showed a reduction in the expression of the fear-related component between phases 1 and 2, *t*_(149)_ = 8.90, *p* < 0.001, *d* = 0.726, and again between phases 2 and 3, *t*_(149)_ = 7.12, *p* < 0.001, *d* = 0.582, indicating a downwards progression of fear-related affective states across phases. Other negative affective states related to the pandemic were not affected by phase, *F*_(2,298)_ = 0.88, *p* = 0.426, $n_{p}^{2}$ = 0.006. Positive affective states related to the pandemic were affected by phase, *F*_(2,298)_ = 5.11, *p* = 0.007, $n_{p}^{2}$ = 0.033. *Post-hoc* comparisons only revealed lowered intensity of positive affective states in phase 3 compared to phase 1, *t*_(149)_ = 3.10, *p* = 0.002, *d* = 0.253. However, the linear contrast was significant, *F*_(1,149)_ = 9.58, *p* = 0.002, $n_{p}^{2}$ = 0.060, indicating a general downwards progression of the intensity of positive affective states related to the pandemic across phases.

**Figure 2:**
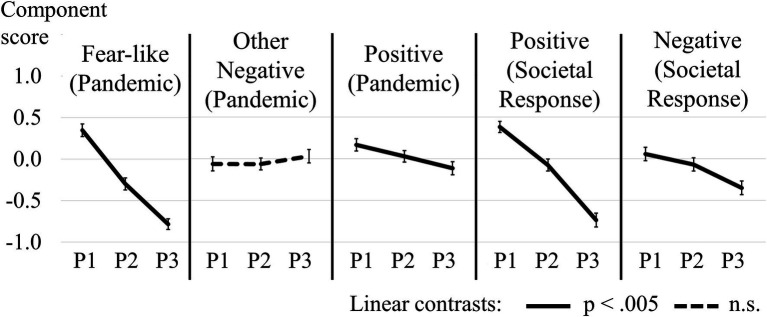
Progression of mean component scores for the intensity of each measured affective state related to the pandemic and the societal response. At the beginning (P1), peak (P2), and aftermath (P3) of the first wave of the pandemic. Full lines show a significant linear contrast, while the dotted line identifies a non-significant linear contrast. Error bars are standard errors of the mean.

Positive affective states related to the societal response differed across phases, *F*_(2,288)_ = 94.47, *p* < 0.001, $n_{p}^{2}$ = 0.396. *Post-hoc* comparisons revealed lower expression of positive affective states related to the societal response at phase 2 compared to phase 1, *t*_(145)_ = 5.79, *p* < 0.001, *d* = 0.496, and lower expression again at phase 3 compared to phase 2, *t*_(145)_ = 8.18, *p* < 0.001, *d* = 0.607, indicating a downwards progression of these affective states across phases. Negative affective states related to the societal response also showed differences across phases, *F*_(2,288)_ = 10.51, *p* < 0.001, $n_{p}^{2}$ = 0.068. *Post-hoc* comparisons only showed differences between phases 2 and 3, *t*_(145)_ = 3.12, *p* = 0.002, *d* = 0.259, and phases 1 and 3, *t*_(145)_ = 4.51, *p* < 0.001. *d* = 0.843. However, the linear contrast was significant, *F*_(1,144)_ = 10.51, *p* = 0.001, $n_{p}^{2}$ = 0.124, suggesting a general downwards progression of the intensity of these affective states across phases. Figure 2 illustrates the progression of mean component scores across the three phases of the study.

### Affective States and Public Health Measures: Within Phases

For data from the beginning of the first wave of the pandemic, a multiple linear regression was calculated to predict compliance with public health measures with sociodemographic information. A significant regression equation was found, *F*_(3,503)_ = 3.95, *p* = 0.008, *R^2^_Adj_* = 0.017. Female gender was the only predictor of higher compliance with public health measures, *β* = 0.149, *p* = 0.001, with no significant effects of age or level of education. We then computed the model again, adding all five components derived from affective state measures. A significant regression equation was once again found, *F*_(8,498)_ = 3.67, *p* < 0.001, *R^2^_Adj_* = 0.040. After adding affective state components to the model, the comparison between both models showed a significant difference, *F*_(5,498)_ = 3.44, *p* = 0.005, confirming higher predictive value when affective states were included as predictors. More intense fear-related affective states related to the pandemic predicted more compliance with public health measures, *β* = 0.169, *p* = 0.002. Female gender remained a significant predictor of higher compliance with public health measures in the second model, *β* = 0.110, *p* = 0.016.

For phase 2 data, a significant regression equation was also found in the regression with sociodemographic predictors of compliance with public health measures, *F*_(3,213)_ = 4.12, *p* = 0.007, *R^2^_Adj_* = 0.042. As in phase 1 data, female gender was the only significant predictor of higher compliance with public health measures, *β* = 0.204, *p* = 0.003. After adding affective state components to the model, a significant regression equation was found again, *F*_(8,208)_ = 3.40, *p* = 0.001, *R^2^_Adj_* = 0.082. The comparison between both models showed a significant difference, *F*_(5,208)_ = 2.86, *p* = 0.016, confirming higher predictive value when affective states were included as predictors. A higher intensity of fear-related affective states related to the pandemic, *β* = 0.163, *p* = 0.036, a higher intensity of positive affective states related to the societal response, *β* = 0.153, *t* = 2.04, *p* = 0.043, and female gender, *β* = 0.154, *p* = 0.043, contributed to the prediction of higher compliance with public health measures in the second model for Phase 2 data.

The multiple regression predicting compliance with public health measures in Phase 3 from sociodemographic factors did not yield a significant regression equation, *F*_(3,159)_ = 2.39, *p* = 0.071, *R^2^_Adj_* = 0.025. Accordingly, no significant predictor of compliance with public health measures was found. However, after adding components derived from the measure of affective states to the model, a significant regression equation was found, *F*_(8,154)_ = 3.79, *p* < 0.001, *R^2^_Adj_* = 0.121. A comparison between models showed higher predictive power for the second regression, *F*_(8,154)_ = 0.4.47, *p* = 0.001. Fear-related affective states related to the pandemic were the only retained predictor of compliance with public health measures in the second model, *β* = 0.377, *t* = 3.47, *p* < 0.001.

## Discussion

We undertook an investigation of pandemic-related affective states, beginning 1 week after the beginning of lockdown in the province of Quebec. We asked responders to report their affective states and again at the peak and the aftermath of the first wave of the COVID-19 pandemic. Thus, we were able to study the variation in the intensity of these affective states across time. Furthermore, we also investigated whether self-reported affective states contributed to the prediction of compliance with public health measures throughout the pandemic.

### The Intensity of Affective States Peaked Near the Onset of the First Wave of the Pandemic

For most affective states, we observed a peak of self-reported intensity at the beginning of the pandemic. The reduction in the intensity of affective states with time was most evident for fear-related affective states related to the pandemic and positive affective states related to the societal response. This result is consistent with research showing a decrease in depressed moods and anxiety symptoms as the first wave of the pandemic progressed (Betini et al., 2021; Fancourt et al., 2021), and less consistent with previous research showing little progression of mood and mental health symptoms through time (Hyland et al., 2020; Martín-Brufau et al., 2020; McPherson et al., 2021). Our data is also not consistent with the idea that the valence of affective states evoked by the pandemic may have shifted from negative to positive, as indicated by social media sentiment analysis (Kruspe et al., 2020; Zhao et al., 2020). Rather, our analyses suggest that positive and negative affective states decreased in intensity over time as the pandemic continued. These results could be interpreted in support of an affective habituation to the pandemic (Frijda, 1988).

However, other explanations could be proposed. For instance, it is possible that these crisis-related affective states were mostly anticipatory and that they subsided as uncertainty about the future of the pandemic was reduced. Supporting this point of view, a timeline of the COVID-19 pandemic in Quebec compiled by the provincial public health organization (Institut national de la santé publique du Québec, 2021b) shows a rapid increase in cases and the implementation of stricter measures in March, when the first phase of our study took place. In contrast, the second phase of our study took place in April, when the number of daily cases and deaths slowly increased to reach a peak. At that time, some public health measures were maintained or made stricter while others were eased. The third phase of our study occurred in June, following a reduction in daily infections and deaths and a general easing of public health measures. A graphical representation of the pandemic’s evolution in the province of Quebec is available in Figure 2. It is possible that participants believed the pandemic to be potentially catastrophic in phase 1, saw the pandemic not accelerating anymore in phase 2, and believed it to be mostly under control in phase 3. Thus, while our data is consistent with affective habituation to the pandemic, affective states may also have followed people’s appraisal of the pandemic’s evolution, with gradual reductions in intensity as the most catastrophic scenarios became increasingly unlikely.

### Affective States Predict Compliance With Public Health Measures

In all three phases of our study, we compared two predictive models of compliance with public health measures. The first model attempted to predict compliance with public health measures from age, gender and level of education. The second model included all of these as predictors, plus components derived from a questionnaire on current affective states. In all three phases of the study, the inclusion of affective states as predictors improved the prediction of compliance with public health measures. In particular, fear-related affective states were the strongest predictor of compliance with public health measures at the beginning, at the peak, and in the aftermath of the first wave of the pandemic. Responders who reported more fear, more anxiety, more shock and a lower feeling of security evoked by COVID-19 reported higher compliance with public health measures.

Positive affective states evoked by the societal response (i.e., the way governments, public health organizations and individuals reacted toward the pandemic) also helped predict compliance with public health measures, but only at the peak of the first wave of the pandemic, not at its beginning or in its aftermath. Higher levels of these affective states were associated with higher compliance with public measures. This result was consistent with other results showing that positive affective states evoked by the societal response modulate reactions to public health campaigns (Bogg and Milad, 2020).

Our results were entirely consistent with the approach-avoidance model (Elliot et al., 2013) which proposes that affective states influence behavior by providing motivation toward approaching some stimuli and avoiding others. Interpreted in the context of this model, our results suggest that people who experienced more negative affective states evoked by the pandemic adopted more behaviors aiming to avoid the disease, and that participants who experienced more positive affective states toward the societal response contributed more readily to this response. Moreover, the stability of the observed statistical link between fear and compliance with public health measures suggests that the intensity of fear-related affective states will likely remain an important predictor of behavior in future global health crises.

### Limits

The current study has its limitations. Of course, our data linking affective states such as fear and anxiety with pandemic-related behavior remain correlational and do not demonstrate a causal relationship between affective states and behavior. One possible way of pushing toward a better understanding of the causal structure linking affective states and behavior in future research would be to use structural equation models, such as the random-intercepts cross-lagged panel model (Hamaker et al., 2015), to investigate whether changes in affective states precede or follow changes in behavior. As we had a relatively low number of participants in the current study, the ratio of data points to parameters fell under an acceptable level for structural equations, and we could not apply this model to our data without risk of overfitting.

Moreover, our sample’s representativeness is limited by a convenience sampling procedure and by a high rate of attrition. Our convenience sample was younger, more educated, and contained a higher proportion of women than the general population of Quebec from which we drew our sample. Other research undertaken simultaneously to our project has suggested that older age (Brouard et al., 2020; Haischer et al., 2020; Solomou and Constantinidou, 2020) and female gender (Galasso et al., 2020; Haischer et al., 2020; Solomou and Constantinidou, 2020) can positively affect compliance with public health measures. We replicated the link between compliance with public health measures and gender in phase one of our study and obtained a similar trend in phase 2, suggesting similarities between our sample and the nationally representative samples used in some other investigations. However, the non-representativeness of the sample may explain why age did not provide predictive value in our statistical models.

An additional limit of our study is that our affective states questionnaire was not exhaustive, and only documented the progression of affective states that were judged most relevant to the pandemic. The inclusion of measures of affective states such as joy and sadness in the questionnaire could have provided a more accurate description of affective states experienced during the pandemic.

## Conclusion

Our research confirms that self-reported affective states related to the pandemic were most intense at the beginning of the pandemic. This finding has theoretical implications for models of the temporal course of affective states and potential practical implications for crisis management research. Our research also underlines the importance of fear-related affective states related to the pandemic and positive affective states related to the societal response as predictors of compliance with public health measures. Future directions for research on psychological determinants of behavior in times of crisis may attempt to directly test models proposing that affective states play a causal role in influencing behavior in times of crisis and, if so, investigate whether they act as a mediator between socio-economic variables and behavior. Future research may also attempt to integrate other variables to models linking affective states and behavior in times of crisis such as exposure to misinformation, personality traits, trust in science, perceived vulnerability, and self-efficacy.

## Data Availability Statement

The raw data supporting the conclusions of this article will be made available by the authors, without undue reservation.

## Ethics Statement

The studies involving human participants were reviewed and approved by Human Subjects Research Ethics Commitee at Université du Québec à Trois-Rivières. The participants provided their written informed consent to participate in this study.

## Author Contributions

YL-S, M-EG, and IB contributed to the conception and design of the study and manuscript revision. YL-S performed the statistical analyses and wrote the first draft of the manuscript. All authors contributed to the article and approved the submitted version.

## Funding

This research was supported by NSERC Discovery Grant 2019–06384 attributed to IB.

## Conflict of Interest

The authors declare that the research was conducted in the absence of any commercial or financial relationships that could be construed as a potential conflict of interest.

## Publisher’s Note

All claims expressed in this article are solely those of the authors and do not necessarily represent those of their affiliated organizations, or those of the publisher, the editors and the reviewers. Any product that may be evaluated in this article, or claim that may be made by its manufacturer, is not guaranteed or endorsed by the publisher.
